# Computational modeling of superparamagnetic nanoparticle-based (affinity) diagnostics

**DOI:** 10.3389/fbioe.2024.1500756

**Published:** 2024-12-06

**Authors:** Loïc Van Dieren, Antoine A. Ruzette, Vlad Tereshenko, Haïzam Oubari, Yanis Berkane, Jonathan Cornacchini, Filip Thiessen EF, Curtis L. Cetrulo, Korkut Uygun, Alexandre G. Lellouch

**Affiliations:** ^1^ Center for Engineering in Medicine and Surgery, Massachusetts General Hospital, Harvard Medical School, Boston, MA, United States; ^2^ Vascularized Composite Allotransplantation Laboratory, Massachusetts General Hospital, Harvard Medical School, Boston, MA, United States; ^3^ Division of Plastic and Reconstructive Surgery, Massachusetts General Hospital, Harvard Medical School, Boston, MA, United States; ^4^ Shriners Children’s Boston, Boston, MA, United States; ^5^ Faculty of Medicine and Health Sciences, University of Antwerp, Wilrijk, Belgium; ^6^ Department of Systems Biology, Harvard Medical School, University of Rennes, Boston, MA, United States; ^7^ Department of Plastic, Reconstructive and Aesthetic Surgery, CHU Rennes, University of Rennes, Rennes, France; ^8^ Gynaecological Oncology Unit, Department of Obstetrics and Gynaecology, Multidisciplinary Breast Clinic, Antwerp University Hospital, University of Antwerp, Antwerp, Belgium; ^9^ Department of Plastic, Reconstructive and Aesthetic Surgery, Multidisciplinary Breast Clinic, Antwerp University Hospital, Antwerp, Belgium; ^10^ Department of Plastic, Reconstructive and Aesthetic Surgery, Ziekenhuis Netwerk Antwerpen, Antwerp, Belgium; ^11^ Division of Plastic and Reconstructive Surgery, Cedars Sinai Medical Center, Los Angeles, CA, United States; ^12^ Unité Mixte de Recherche UMR 1236 Suivi Immunologique des Thérapeutiques Innovantes, INSERM and University of Rennes, Rennes, France; ^13^ Unité Mixte de Recherche UMR-S 1140 Innovative Therapies in Haemostais, INSERM and University of Paris, Paris, France

**Keywords:** coil, COMSOL, diagnostic, iron oxide, magnetic nanoparticles, superparamagnetism

## Abstract

**Introduction:**

Magnetic nanoparticles (MNPs), particularly iron oxide nanoparticles (IONPs), are renowned for their superparamagnetic behavior, allowing precise control under external magnetic fields. This characteristic makes them ideal for biomedical applications, including diagnostics and drug delivery. Superparamagnetic IONPs, which exhibit magnetization only in the presence of an external field, can be functionalized with ligands for targeted affinity diagnostics. This study presents a computational model to explore the induced voltage in a search coil when MNPs pass through a simulated blood vessel, aiming to improve non-invasive diagnostic methods for disease detection and monitoring.

**Methods:**

A finite element model was constructed using COMSOL Multiphysics to simulate the behavior of IONPs within a dynamic blood vessel environment. Governing equations such as Ampère’s law and Faraday’s law of induction were incorporated to simulate the induced voltage in a copper coil as MNPs of various sizes flowed through the vessel. Rheological parameters, including blood viscosity and flow rates, were factored into the model using a non-Newtonian fluid approach.

**Results:**

The amount of MNPs required for detection varies significantly based on the sensitivity of the detection equipment and the size of the nanoparticles themselves. For highly sensitive devices like a SQUID voltmeter, with a coil sensitivity approximately 10^−12^ V, very low MNP concentrations—approximately 10^−4^ μg/mL—are sufficient for detection, staying well within the safe range. As coil sensitivity decreases, such as with standard voltmeters at 10^−8^ V or 10^−6^ V, the MNP concentration required for detection rises, approaching or even exceeding potentially toxic levels. Additionally, the physical size of MNPs plays a role; larger nanoparticles (e.g., 50 nm radius) require fewer total particles for detection at the same sensitivity than smaller particles like those with a 2.5 nm radius. For instance, at a coil sensitivity of 10^−10^ V, a 2.5 nm particle requires approximately 10^12^ particles, whereas a 50-nm particle only needs 10^8^. This highlights the importance of optimizing both detection sensitivity and particle size to balance effective detection with safety.

**Conclusion:**

This computational model demonstrates the feasibility of using superparamagnetic nanoparticles in real-time, non-invasive diagnostic systems.

## 1 Introduction

Magnetic nanoparticles (MNPs) are small-sized particles, typically ranging from 1 nm to 100 nm (Mittal et al., 2022). This size range offers unique capabilities for non-invasive manipulation within the human body using external magnetic fields (Murzin et al., 2020). However, their suitability for biomedical applications depends on various factors, including their chemical composition, size, shape, morphology, and magnetic behavior (Wu et al., 2019). Biocompatible coatings are essential to ensure MNPs can safely integrate into biological systems (Vangijzegem et al., 2019). These coatings not only enhance biocompatibility but also modify the magnetic properties of MNPs and facilitate their interaction with specific ligands. By altering surface chemistry, MNPs can acquire multifunctionality. This allows diverse applications such as combined hyperthermia–drug delivery, magnetic neuromodulation, multimodal imaging, and *in vitro* diagnostics, including cell sorting and diagnosis (Dadfar et al., 2019; Farzin et al., 2020; Hilger, 2013; Schaller et al., 2009; Koh and Josephson, 2009). However, challenges in MNP-based diagnostics limit their clinical utility. A primary concern is the detection limit, as current techniques often have low sensitivity, making it difficult to detect biomarkers at low concentrations critical for early diagnosis (Balaban Hanoglu et al., 2023; Su et al., 2020; Farinha et al., 2021). The toxicity of MNPs depends on factors like structural properties, dosage, and chemical composition; while some materials, inert in bulk, become toxic at nanoscale (e.g., gold), metals like cobalt and cadmium are harmful at all scales, whereas titanium and iron oxide are generally safer for cells (Markides et al., 2012). Currently, MNPs are predominantly used *in vivo* to enhance MRI contrast through improved relaxation times (Yu et al., 2011). However, another potential lies in their ability to selectively bind to a variety of molecular targets (Malekzad et al., 2017; Ren et al., 2020).

Iron oxide nanoparticles (IONPs) exhibit superparamagnetic behavior, a property crucial for their use in biomedical and technological applications (Wu et al., 2019; Maldonado-Camargo et al., 2017). IONPs display unique magnetic characteristics that differ from bulk iron oxide (Peng et al., 2006). While bulk iron oxide is ferromagnetic and retains magnetic properties even after removing an external magnetic field, iron oxide nanoparticles exhibit superparamagnetism (Patsula et al., 2016). This phenomenon occurs because, at small sizes, thermal energy can easily overcome the magnetic anisotropy energy barrier, causing the magnetic moments within the nanoparticles to randomly fluctuate and align with an external magnetic field when applied (Issa et al., 2013). Once the external field is removed, these nanoparticles quickly lose their magnetization. This reversible behavior makes them useful for applications where controlled and transient magnetic responses are desired, such as in targeted drug delivery systems, MRI contrast agents for enhanced diagnostic imaging, hyperthermia therapy for cancer treatment, and even in environmental remediation processes.

Additionally, the superparamagnetic behavior of IONPs allows them to induce a voltage in a coil when excited by an alternating current (AC) field; this happens through a changing magnetic flux (Du et al., 2022; Noguchi et al., 2022). The concept of magnetic flux is fundamental to understanding electromagnetic induction, where changes in magnetic flux through a surface induce an electromotive force (EMF), or voltage, in a nearby coil (Kinsler, 2020; Sears, 1963). Magnetic flux, denoted by Φ, represents the measure of magnetic field lines passing through a given area. It is defined mathematically as Φ = B × A × cos(θ), where B is the magnetic field strength, A is the area perpendicular to the magnetic field, and θ is the angle between the magnetic field and the normal to the surface (Romer, 1982). According to Faraday’s law of electromagnetic induction, any change in magnetic flux through a coil of wire induces a voltage across its ends.

## 2 Hypothetical

**FIGURE 1:**
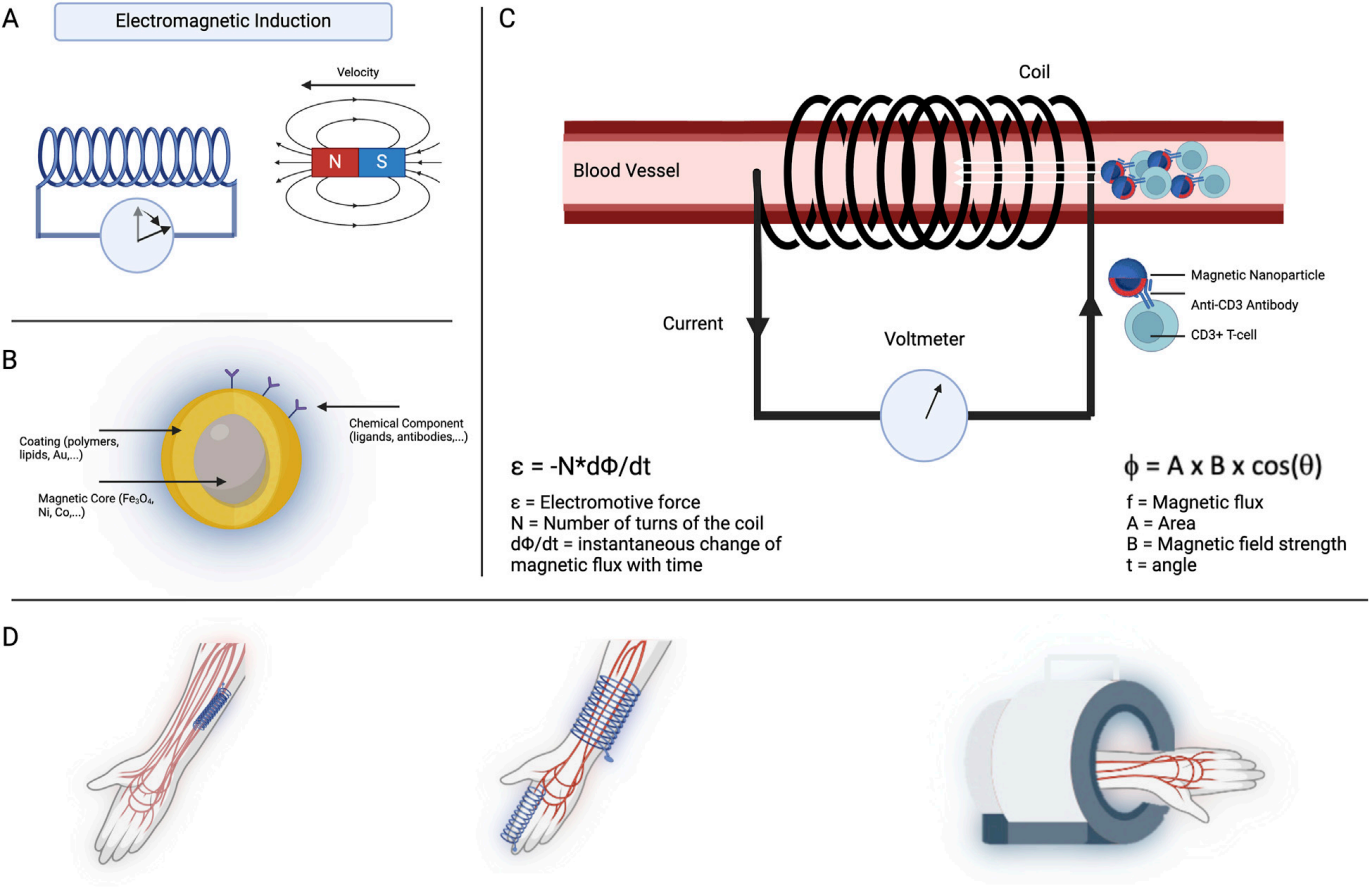
Concept illustration. **(A)** Faraday’s law of electromagnetic induction. As a magnet moves through or toward a coil, voltage is induced inside the coil. When the magnet is static, no change in magnetic flux occurs, and therefore, the voltmeter rests at 0, as shown by the first state of the arrow. However, when a movement is induced in the magnet, the magnetic flux changes, and a voltage is induced in the coil, as evidenced by the movement of the arrow. **(B)** A single magnetic nanoparticle is usually composed of three different domains: a magnetic core, a coating, and a chemical component. The magnetic core is responsible for the magnetic properties attributed to the particle. The coating serves for biocompatibility purposes and allows complexing with chemical components. Those chemical components can be functional ligands such as antibodies. **(C)** In this concept illustration, magnetic nanoparticles are injected into the circulatory system of the patient. Chemical components attached to the MNP’s surface trigger binding to a specific target cell or marker of interest (i.e., T cells). Once bound, the MNP–target complex induces a distinct voltage profile inside the coil. The dynamic magnetic flux within the search coil generates an electromotive force, which can be detected using a voltmeter. In this image, the coil surrounds the vessel. However, we could easily imagine a wristband or peribrachial coil **(D)**.

We present the concept of a diagnostic platform that detects and monitors MNPs in the bloodstream by using external magnetic fields to induce measurable voltage changes inside a coil (Figure 1). This capability forms the foundation of a sensitive and specific diagnostic tool. If particles are not functionalized with a ligand, they could provide information about renal and/or hepatic clearance, depending on their size; they could also inform about blood rheological parameters. Alternatively, particles functionalized with specific ligands could assess a diverse array of biomarkers. The device’s versatility extends to its application methods: it can be placed invasively around a vessel for precise monitoring or noninvasively as a ring or wristband for continuous monitoring. Alternatively, it can be bulkier as a portable device, providing enhanced sensitivity for detailed diagnostics. The real-time data integration capabilities of this device would offer healthcare providers immediate feedback on disease progression, treatment efficacy, or overall patient health.

## 3 Methods

### 3.1 Magnetic properties of iron oxide and magnetic field strength

#### 3.1.1 Numerical model

A finite element numerical model was developed using COMSOL Multiphysics (version 5.5, Stockholm, Sweden). This model utilized the magnetic fields (mf) module to explore the material properties of iron oxide and cobalt ferrite, as well as to analyze the magnetic field strength generated by a coil.

#### 3.1.2 Governing equations

In this study, the governing Equations 1–6 for the magnetic and electric fields, as well as the current densities, are fundamental to our modeling approach. The magnetic field intensity *H* is related to the current density *J* through Ampère’s Law:

∇×H=J, (1)

where ∇ × H represents the curl of the magnetic field and *J* is the current density. The magnetic flux density *B* is derived from the magnetic vector potential *A* as follows:

B=∇×A . (2)

Here, *B* denotes the magnetic flux density, and *A* is the magnetic vector potential. The total current density *J* consists of the conduction current density and any external current density *J*_*e*_, expressed as

$$J=\sigma E+J_{e,}$$ (3)

where σ is the electrical conductivity, E is the electric field intensity, and $J_{e}$ is the external current density. The external current density $J_{e}$ is given by

$$J_{e}=\frac{NI_{coil}}{A}e_{coil},$$ (4)

where N is the number of turns inside the coil, $I_{coil}$ is the current through the coil, A is the cross-sectional area of the coil, and $e_{coil}$ is the unit vector in the direction of the coil. The electric field E is related to the magnetic vector potential A through the time-derivative.

$$E=-\frac{\partial A}{\partial t}.$$ (5)

#### 3.1.3 Geometry and material properties

A copper coil (5 cm × 5 cm) with 40 turns was modeled in COMSOL to generate a magnetic field capable of inducing magnetization in a material placed at its center. The current induced in the coil was set at 5000 A at 50 Hz to allow the material to reach its saturation. The material brought to saturation and their properties (Table 1) are governed by the Jiles–Atherton hysteresis, where

$$B=\mu_{0}\left(H+M\right),$$ (6)

**TABLE 1:** Material properties of iron oxide and cobalt ferrite.

Property	Unit	Value for iron oxide	Value for cobalt ferrite
Electrical conductivity	S/m	0.13	5.20e6
Relative permittivity	1	1.00	1.00
Saturation magnetization	A/m	2.88e5	2.40e7
Domain wall density	A/m	1.00e5	1.00e5
Pinning loss	A/m	5.00e5	2.00e5
Magnetization reversibility	1	0.95	0.4
Interdomain coupling	1	0.5	1.4

and where $\mu_{0}$ is the permeability of free space, H is the magnetic field intensity, and M is the magnetization of the material.

For the magnetic field induced by a coil, the coil was powered by an AC from a standard outlet with a voltage of 220 V and a frequency of 50 Hz. The coil’s radius and height were set at 5 cm and 10 cm, respectively.

### 3.2 Induced voltage in a search coil by a single MNP

A 2D computational simulation was performed using COMSOL Multiphysics to evaluate the induced voltage by a single MNP moving within a blood vessel. The simulation was built on the same governing equations as in Section 3.1. Additionally, we accounted for the rheological properties of blood by utilizing an inelastic non-Newtonian fluid model based on the Carreau model. The model parameters used were zero shear rate viscosity (0.056 Pa·s), infinite shear rate viscosity (0.0035 Pa·s), time constant (3.313 s), and power-law index (0.3568). A laminar flow inlet was created with a velocity of 7.1 cm/s, allowing the MNP to move freely due to the coupling between the laminar flow and solid structure modules. The particle’s motion within the flow was simulated at different points in time to capture the magnetization profile of MNPs with varying diameters (5 nm, 30 nm, 50 nm, and 100 nm).

### 3.3 Induced voltage in a search coil by multiple MNPs

The flux inside the coil was first calculated for a single particle using the following formula (Tu, 2013):

$$\emptyset_{0}=\int B\cdot ds=\frac{\mu_{0}m}{4\pi }\overset{2\pi }{\underset{0}{\int }}\overset{\infty }{\underset{R}{\int }}\frac{1}{r^{3}}r\;drd\theta ,$$ (7)

where ( ∫B⋅ds ) is the line integral of the magnetic field (B) along the path of the current loop. It is integrated over the entire loop, and it represents the total magnetic flux through the loop. μ_0_ represents the permeability of free space, which is a constant in electromagnetism (4π x 10^−7^ Tm/A), *m* represents the magnetic moment of a single 30-nm iron oxide MNP, and 4π is related to the spherical geometry of the problem. The double integral represents the contribution of the magnetic field due to the current loop at a point in space. The outer integral is with respect to the angle (θ) around the loop, and the inner integral is with respect to the radial distance (*r*) from the center of the loop. The integrand $\frac{1}{r^{3}}$ is related to the magnetic field expression for a magnetic dipole.

$$v\left(t\right)=n\frac{d\left(\emptyset \left(t\right)\right)}{dt}=n\frac{d\left(\emptyset_{0}\times \cos\left(\omega t\right)\right)}{dt}.$$ (8)

According to Faraday’s law of induction, the induced voltage in the coil can be calculated as shown in Equation 8. v(t) is the expression of voltage over time, *n* represents the number of turns in the coil, and $\frac{d\left(\emptyset \left(t\right)\right)}{dt}$ expresses the change of magnetic flux over time, with ϖ being the angle between the magnetic vector of the MNP and the coil. The distance to the center of the coil in the *x*-axis, calculated to determine the motion of the particle (hence, the magnetic flux), was calculated using the following formula:

$$distance_{to_{center}}=\sqrt{{coordinate_{X}}^{2}+\left|coil_{radius}+{coordinate_{Y}}^{2}+{coordinate_{Z}}^{2}\right|.}$$ (9)

Equations 7–9 were implemented into Blender (version 4.2.0., Amsterdam, Netherlands), and the simulations were performed using a bidirectional cylindrical geometry using the particle generator module. The same equations were also implemented in Jupyter Notebook (Project Jupyter) to calculate the toxicity range of the particles.

### 3.4 Determining the safe amount of MNPs for detection

The detection limits of MNPs were assessed using different voltmeters, including a SQUID voltmeter, a Keithley Nanovoltmeter Model 2182A, and a Keithley DMM6500 6.5. The relationship between coil sensitivity and the number of MNPs required to induce a detectable voltage signal was modeled. The detection limit was determined for different MNP sizes (5 nm, 30 nm, 50 nm, and 100 nm) and compared to safe MNP concentrations. The model considered physiological parameters such as cardiac output (5 L/min), heart rate (70 bpm), stroke volume (71.4 mL), and the percentage of cardiac output directed to the forearm (2.3%) and leg (6.5%). The total blood volume per arterial pulse reaching the forearm and leg was calculated to be approximately 1.5 mL and 4.5 mL, respectively. The voltage induced by MNPs within these volumes was then simulated, providing insight into the safe ranges for MNP doses in biomedical applications.

## 4 Results

### 4.1 Magnetic properties of iron oxide nanoparticles

**FIGURE 2:**
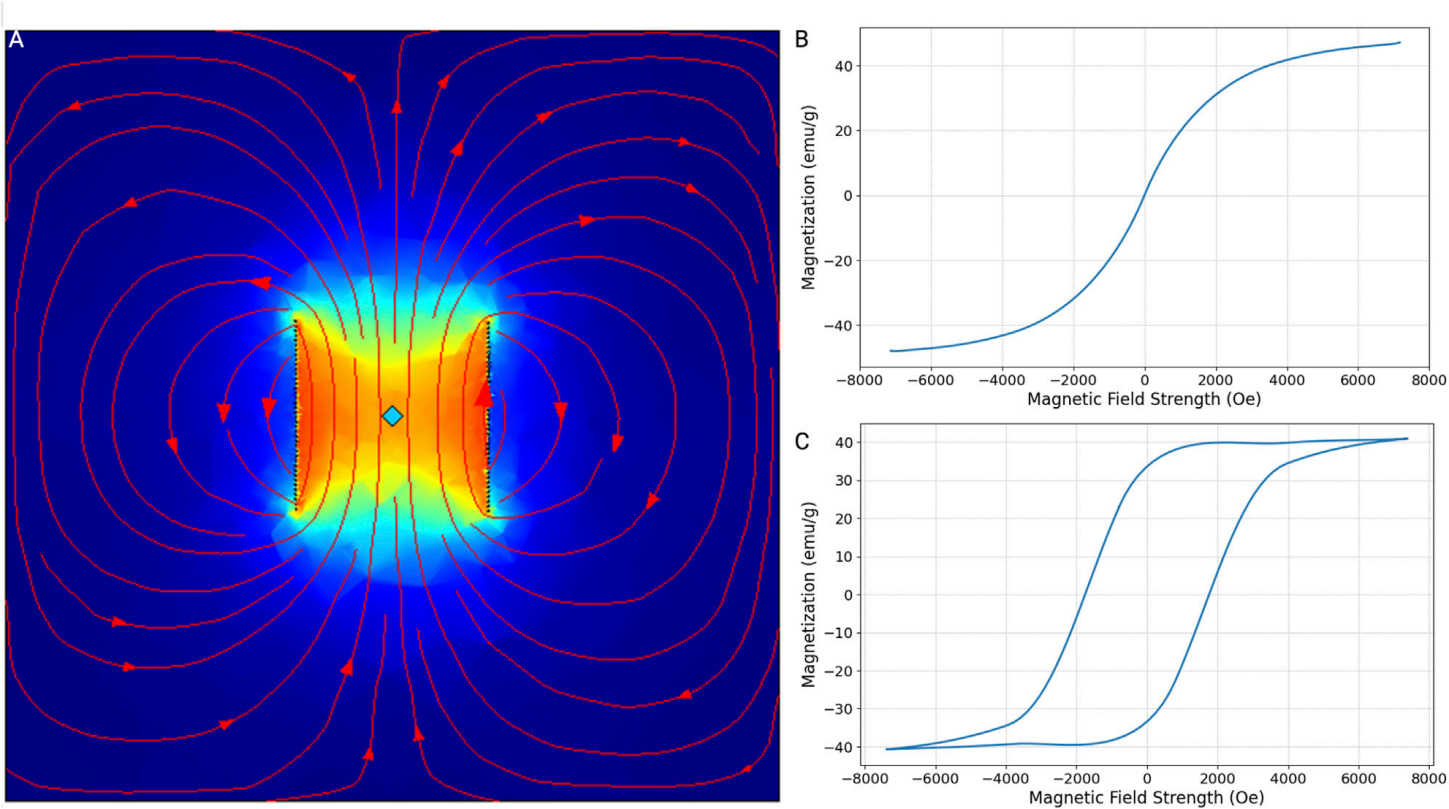
Magnetization-magnetic field strength curves (M-H curves). **(A)** This plot illustrates the magnetic field strength of a 5-cm by 5-cm copper coil consisting of 40 turns. The material at the center of the coil has reached its saturation magnetization due to the current induced within the coil. The field lines and intensity indicate the distribution and magnitude of the magnetic field, showing how the material interacts with the magnetic field generated by the coil. M-H curves were simulated for iron oxide and cobalt ferrite using this model. **(B)** Iron oxide, particularly in its nanoparticle form, exhibits superparamagnetic behavior. This means that in the absence of an external magnetic field, the material does not retain any magnetization. However, when an external magnetic field is applied, the magnetic moments align with the field, resulting in a high magnetization. As the field strength increases, the magnetization of iron oxide rapidly increases and approaches saturation, but without the hysteresis typical of ferromagnetic materials, indicating no remanent magnetization or coercivity. **(C)** Unlike iron oxide, cobalt ferrite exhibits ferromagnetic properties. It shows an initial rapid increase in magnetization, eventually reaching saturation. The hysteresis loop in the curve indicates significant remanent magnetization and high coercivity.

The magnetic properties of nanoparticles are important to determine their applicability in various technological and biomedical fields. Our 3D model elaborated on the magnetic behavior of superparamagnetic materials (Figure 2A). Briefly, a piece of iron oxide or cobalt ferrite was placed inside a coil. Next, the material was brought to saturation by a coil excited by an alternative current. Iron oxide nanoparticles exhibit superparamagnetic characteristics, as evidenced by their magnetization response to an external magnetic field (Figure 2B). Upon the application of a magnetic field, these nanoparticles align their magnetic moments along the field direction, increasing their magnetization. However, they do not retain any magnetization once the external field is removed, displaying no hysteresis. This behavior is advantageous for our applications, where a quick induction and absence of residual magnetization are essential. In contrast, cobalt ferrite nanoparticles demonstrate a pronounced hysteresis loop in their magnetization curve (Figure 2C). These nanoparticles retain a significant amount of magnetization even after the external field is withdrawn. This indicates ferromagnetic behavior. The coercivity and remanence observed in these nanoparticles make them unsuitable for our application, where ferromagnetic behavior outside the field of the coil could lead to clustering of the MNPs and clotting of the blood vessel.

Using this model, we also determined the maximum magnetic field that could be induced by a coil powered by a standard outlet with a voltage of 220 V and a frequency of 50 Hz. Because the length is directly proportional to its resistance and inversely proportional to current, the number of turns inside the coil does not impact the induced magnetic field. Hence, a coil with n number of turns, a radius of 5 cm, and a height of 10 cm induces a magnetic field of 2299.1 Oe. This, in turn, leads to a magnetization for a single 5 nm, 30 nm, 50 nm, and 100 nm iron oxide MNP of 1.15e−17 emu, 2.3e−15 emu, 12.3.15e−14 emu, and 9.19e−14 emu, respectively.

### 4.2 Induced voltage in a coil by a single MNP

**FIGURE 3:**
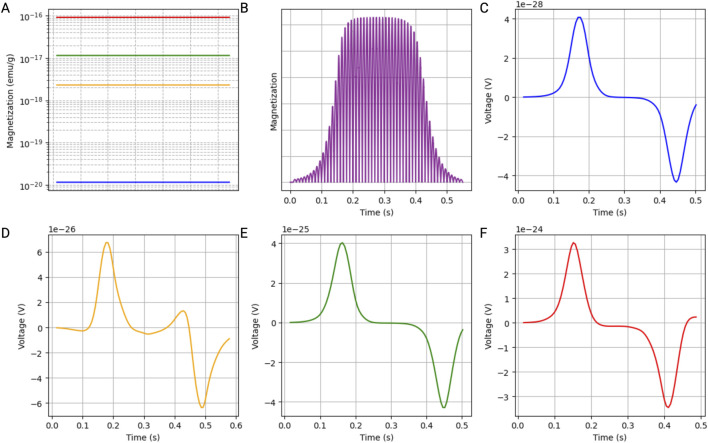
Simulation results of a single iron oxide magnetic nanoparticle (MNP) within a blood vessel. The parameters were selected to accurately represent the rheological properties of blood under physiological conditions. **(A)** Magnetization of the MNPs, illustrating how different MNP sizes (5 nm: blue; 30 nm: yellow; 50 nm: green; 100 nm: red) respond to an alternating magnetic field. Those results were extrapolated from the M-H curves. **(B)** Magnetization profile of the MNPs over time, highlighting the oscillatory behavior induced by the alternating magnetic field, with the magnetization curve indicating the time at which the MNPs travel through the center of the coil. **(C–F)** Detailed voltage measurements over time, captured by the search coil system at different points within the simulated blood vessel, showing the induced signal by a single MNP with a diameter of 5 nm **(C)**, 30 nm **(D)**, 50 nm **(E)**, and 100 nm **(F)**.

The magnetization of the MNPs over time reveals how different MNP sizes respond under varying magnetic field strengths, with color-coded lines illustrating distinct behaviors depending on their size (Figure 3A). Additionally, the magnetization profile of the MNPs depicts their magnetization over time, highlighting the oscillatory behavior induced by the alternating magnetic field. The magnetization curve indicates the time at which the MNPs travel through the center of the coil (Figure 3B). Detailed voltage measurements over time, captured by the search coil system at different points within the simulated blood vessel, inform us about the induced signal by a single 5-nm (Figure 3C), 30-nm (Figure 3D), 50-nm (Figure 3E), or 100-nm (Figure 3F) MNP.

### 4.3 Induced voltage in a coil by multiple MNPs

**FIGURE 4:**
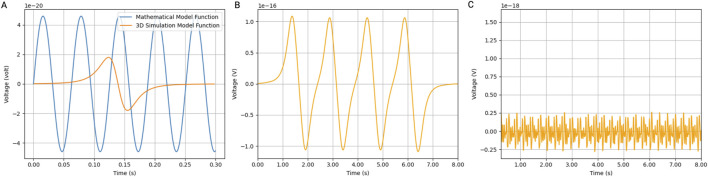
Induced voltage in coil by MNPs. Simulations were performed in Blender using a bidirectional cylindrical geometry and the particle generator module. **(A)** The induced voltage in a coil (with a radius of 5 cm and 2000 turns was simulated) by a 30 nm iron oxide magnetic nanoparticle (IONP) at a velocity of 0.7 m/s compared to the mathematical model function. **(B)** Using the same Blender model, 1,000 magnetic IONPs were generated in pulses at a frequency of 1 Hz. **(C)** The noise produced by the IONPs flowing in the opposite direction. Ten thousand particles flowing at 0.07 m/s were generated in a continuous mode.

To validate the model, the induced voltage of a single MNP was computed and compared to the induced voltage mathematically calculated (Figure 4A). The results show a similar order of magnitude of voltage induced in the coil. The difference can be attributed to particle velocity, which is not accounted for in the mathematical model function.

We also calculated the induced voltage in a coil by a pulsating artery. The induced voltage shows a linear relationship with the number of particles generated per pulse. Figure 4B shows the induced voltage by a thousand particles per pulse in a coil with a radius of 5 cm and 2000 turns. We also considered the particles that flow in the opposite direction of the pulsating artery inside the vein. Therefore, we simulated a continuous flow of 10 times the number of particles at a 10 times lower velocity. The noise created is a couple of orders of magnitudes lower than the signal generated by the arterial pulses (Figure 4C).

**FIGURE 5:**
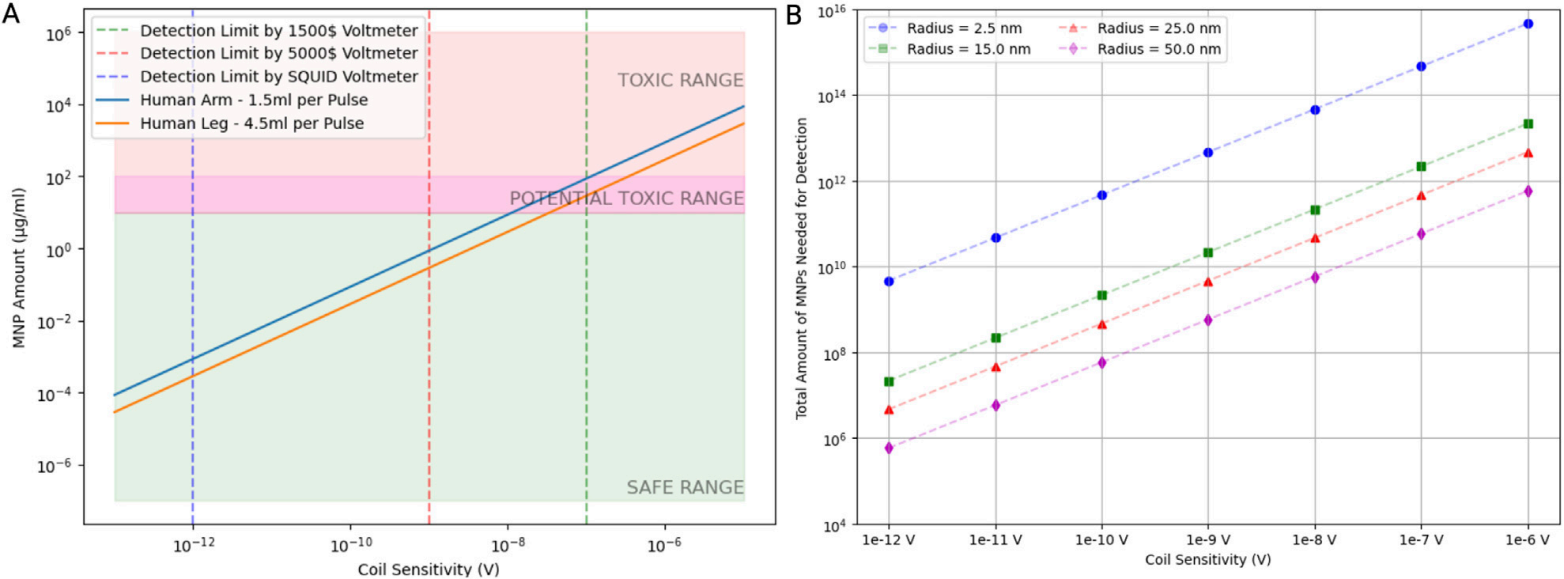
A safe number of magnetic nanoparticles induces a detectable voltage signal. **(A)** The relationship between coil sensitivity and the mass of magnetic nanoparticles (MNPs) required to produce a detectable voltage signal. Detection limits are shown for different voltmeter sensitivities along with the placement of the coil. Assuming one arterial pulse passes through the coil for each timestep, the difference in mass of MNPs needed for detection was calculated for a pulse volume in the forearm and leg, respectively. The green shaded area represents the safe range of the MNP mass, the pink shaded area represents the toxic range, and the transition region is potentially toxic. **(B)** The number of MNPs needed to induce a detectable voltage signal as a function of coil sensitivity for different particle radii. Data points indicate how particle size influences the required MNP quantity.

Lastly, we demonstrate that the detection limits of MNPs vary significantly with the type of voltmeter used (Figure 5). The SQUID voltmeter, with the highest sensitivity, has a detection limit at the lowest MNP concentration, followed by the 5,000$ voltmeter (Keithley Nanovoltmeter Model 2182A, Keithley, Cleveland, Ohio, USA) and the 1,500$ voltmeter (Keithley DMM6500 6.5, Keithley, Cleveland, Ohio, USA). The MNP amounts required for detection fall into the safe range at lower coil sensitivities but move into the potentially toxic range and toxic range as the sensitivity decreases. As coil sensitivity increases, the total amount of MNPs needed for detection decreases. Additionally, the required MNP amount is inversely proportional to the MNP radius: larger MNPs require fewer particles for detection at a given sensitivity. This indicates that for applications requiring high sensitivity, using larger MNPs could be advantageous in minimizing the total nanoparticle load while maintaining detectability. These findings are crucial for optimizing the use of MNPs in biomedical applications. Ensuring MNP amounts remain within the safe range while achieving sufficient detectability is vital for minimizing toxicity risks. Advanced detection methods like SQUID voltmeters offer significant advantages in sensitivity, allowing for lower MNP doses and reducing potential toxicity.

## 5 Discussion

We present the concept of a nanoparticle-based affinity detection platform for detecting circulating MNPs, enabling clinicians to monitor and diagnose multiple conditions using a single device and pre-complexed MNPs. The perivascular coil detects magnetic field variations from MNPs in the pulsatile bloodstream, where these MNPs, selectively binding to disease or drug targets, act as molecular probes (Pan et al., 2012; Veiseh et al., 2010). This approach addresses challenges in current diagnostics by facilitating real-time monitoring of MNP concentrations, thereby potentially increasing diagnostic resolution. Our simulation indicates that noise generated by venous MNP flow, moving in the opposite direction at a slower rate, is substantially lower than the arterial pulse signal; in practical devices, this noise can be further mitigated with signal processing strategies like targeted filtering and device design like magnetic shielding, supporting the platform’s high sensitivity and specificity.

The versatility of the device implies that a single injection of complexed MNPs could facilitate the sensibility threshold for various conditions, from specific diseases to drug concentrations (Chandrakala et al., 2022). Practical implementation of the perivascular coil involves addressing challenges such as biocompatibility, miniaturization, and sensitivity (Ye and Barrett, 2021). Therefore, alternative designs like a portable magnetic field sensor could be explored. Such sensors, including fluxgate or giant magneto-impedance magnetometers, offer more complex and sensitive detection capabilities (Schoinas et al., 2020; Lu and Huang, 2015). A wearable solution, such as an arm cuff, wristband, or ring, could also be considered (Shi et al., 2022). The simulations demonstrate the potential and feasibility of this platform using a simple search coil. While basic equipment suffices to show the concept, advanced approaches can enhance accuracy and efficacy. Simulations confirm that the platform operates safely within non-toxic parameters. Current simulations use a 220 V, 50 Hz power outlet, but alternative solutions could increase particle magnetization, further improving the platform’s potential applications.

When considering the diagnostic potential of MNPs for *in vivo* applications, the ability to differentiate between bound and unbound nanoparticles is critical (Niemirowicz et al., 2012). Achieving this distinction is needed for the precision and reliability of diagnostic outcomes because only bound MNPs will accurately reflect the presence of the target. A possible strategy for distinguishing between particles that have found their target and those still in circulation involves exploring two potential avenues. First, considering the body’s natural clearance mechanisms, bound MNPs might exhibit a distinct clearance rate compared to their unbound counterparts (Zelepukin et al., 2020). This difference in how quickly they leave the body could offer a valuable time window for monitoring the presence of MNPs attached to specific targets. Additionally, another avenue could be investigating the electrical signals generated by the MNPs as they move through a coil system (Kim et al., 2020). The interaction between the bound and unbound MNPs with the coil might yield a distinct voltage profile based on the drag forces experienced by the bound and unbound particles. If successful, this approach could provide a real-time and non-invasive means of differentiating between particles based on their magnetic behavior. Further research and experimentation are essential to validate and refine these hypotheses for practical application.

A first application for this diagnostic platform may be calculating clearance and determining the rheological parameters of the circulatory system. This method simplifies diagnostics by eliminating the need to distinguish between bound and unbound MNPs (Charlton et al., 2013). In chronic kidney disease (CKD), accurate assessment of glomerular filtration rate (GFR) is crucial. Monitoring MNP clearance rates offers a direct and efficient approach to assessing kidney function. Introducing MNPs into the bloodstream and observing their gradual disappearance provides valuable insights into clearance dynamics, correlating directly with kidney function (Medeiros et al., 2011). This approach not only simplifies the diagnostic procedure but also has the potential to significantly enhance the accuracy of GFR assessments. The need for frequent and sometimes invasive GFR tests, such as after transplantation, could be mitigated, which would improve the patient experience and facilitate more accessible monitoring (Shlipak et al., 2021).

This methodology can be further extended to diagnose target cells bound to MNPs, such as T-cells, in the context of transplantation rejection. By conjugating MNPs with specific ligands or antibodies that bind to T-cells, it becomes possible to track the migration and accumulation of these immune cells in the body in the case of organ transplantation, where the timely detection of transplant rejection is crucial (Lantieri et al., 2020). Additionally, similar strategies could be employed to monitor other cell types implicated in various diseases, such as tracking metastatic cancer cells or identifying infections.

If the detection is sensitive enough to detect a single cell loaded with MNPs, this method could be used to monitor a significantly expanded blood volume. One major limitation of current diagnostic methods is their reliance on small volumes of extracted blood. The current sensitivity level, typically ranging from 1 to 10 cells/mL, translates to only 5,000 to 50,000 cells in the entire blood volume. This low detection threshold is inadequate for timely intervention in diseases such as metastasis, where early detection is critical for effective treatment. Additionally, current *ex vivo* and *in vitro* diagnostic tests require the extraction of cells or markers from living organisms, which can alter their properties and impede their study within a natural biological context. The preparation procedures for *ex vivo* testing can also be time-consuming, lasting hours or even a full day, leading to delays in diagnosis and treatment. *Ex vivo* sampling is typically discontinuous, occurring at discrete time points, which further complicates the detection of dynamic changes in cell populations over time. Addressing these limitations requires the development of novel diagnostic technologies that enable non-invasive, real-time monitoring of cellular dynamics within the body. Such advancements could revolutionize early disease detection and facilitate prompt therapeutic interventions, ultimately improving patient outcomes.

While the proposed diagnostic approaches hold promise, it is crucial to recognize and address several inherent limitations. Biocompatibility stands out as a primary concern. The use of MNPs necessitates careful consideration of potential toxicity associated with the use of MNPs (Malhotra et al., 2020). Ensuring the safety of patients requires a thorough understanding of the long-term effects and clearance mechanisms of MNPs, particularly in continuous monitoring scenarios. The unique properties of MNPs, such as their high surface area-to-volume ratio and magnetic responsiveness, make them ideal candidates for innovative diagnostic applications. However, these same characteristics can also increase the likelihood of potential toxicity. Several factors contribute to this concern, including the size, shape, composition, and surface coatings of MNPs. For instance, while certain coatings can enhance MNP biocompatibility by preventing aggregation and reducing interaction with cellular structures, others might lead to adverse cellular responses if they degrade over time or interact unpredictably with biological molecules (Portilla et al., 2022).

The safety of MNPs is closely tied to our understanding of their long-term effects and clearance mechanisms within the body (Nowak-Jary and Machnicka, 2023). Because MNPs are foreign entities, it is vital to determine how they are metabolized, whether they accumulate in specific organs (such as the liver, spleen, or kidneys), and how the body ultimately clears them. Prolonged retention of MNPs in certain tissues could lead to chronic inflammation, oxidative stress, or other adverse immune responses (Tulinska et al., 2022), particularly in continuous monitoring applications where repeated doses or sustained presence might be necessary. Achieving miniaturization without sacrificing sensitivity poses an engineering challenge that demands rigorous testing and refinement. Furthermore, the sensitivity and specificity of MNPs in binding to targets may introduce variations in diagnostic accuracy. Real-world applications may encounter environmental factors and patient-specific conditions influencing device performance.

The finite element model developed in COMSOL has several inherent limitations, primarily due to simplifications and assumptions that ensure computational feasibility but may reduce model accuracy. One key limitation is the idealization of the coil’s material and geometry; properties like electrical conductivity and magnetic permeability are assumed to be uniform, which may not fully represent real-world variations in material quality or structural inconsistencies. In the rheological modeling of blood, the non-Newtonian Carreau model captures basic flow behavior but does not consider complex interactions with vessel walls or blood cells, potentially affecting the accuracy of nanoparticle motion and magnetic response predictions. Lastly, we acknowledge that the aggregation of MNPs within blood vessels can significantly impact their effective size and detection limits. While our analysis incorporated different particle sizes to account for potential variations, it is important to recognize that computing the precise dynamics of aggregation is a complex challenge. The size changes considered in our analyses represent a simplification of the intricate interactions occurring *in vivo*.

Overall, our findings provide a feasible *in silico* model for utilizing MNPs and a magnetic coil. The calculated parameters offer quantitative measures that guide experimental designs, providing a basis for optimizing MNP concentrations and coil sensitivities in practical applications. Moreover, the presented equations offer a pathway for tailoring detection systems based on specific coil configurations and MNP characteristics, contributing to the advancement of magnetic nanoparticle-based diagnostics. The thorough consideration of these parameters enhances the feasibility and reliability of detecting MNPs in various biomedical applications, laying the groundwork for further investigations and refinements in the field.

## Data Availability

The raw data supporting the conclusions of this article will be made available by the authors, without undue reservation.
