# Corrigendum: Computational modeling of superparamagnetic nanoparticle-based (affinity) diagnostics

**DOI:** 10.3389/fbioe.2025.1610782

**Published:** 2025-05-20

**Authors:** Loï Van Dieren, Antoine A. Ruzette, Vlad Tereshenko, Haïzam Oubari, Yanis Berkane, Jonathan Cornacchini, Filip Thiessen EF, Curtis L. Cetrulo, Korkut Uygun, Alexandre G. Lellouch

**Affiliations:** ^1^ Center for Engineering in Medicine and Surgery, Massachusetts General Hospital, Harvard Medical School, Boston, MA, United States; ^2^ Vascularized Composite Allotransplantation Laboratory, Massachusetts General Hospital, Harvard Medical School, Boston, MA, United States; ^3^ Division of Plastic and Reconstructive Surgery, Massachusetts General Hospital, Harvard Medical School, Boston, MA, United States; ^4^ Shriners Children’s Boston, Boston, MA, United States; ^5^ Faculty of Medicine and Health Sciences, University of Antwerp, Wilrijk, Belgium; ^6^ Department of Systems Biology, Harvard Medical School, Boston, MA, United States; ^7^ Department of Plastic, Reconstructive and Aesthetic Surgery, CHU Rennes, University of Rennes, Rennes, France; ^8^ Gynaecological Oncology Unit, Department of Obstetrics and Gynaecology, Multidisciplinary Breast Clinic, Antwerp University Hospital, University of Antwerp, Antwerp, Belgium; ^9^ Department of Plastic, Reconstructive and Aesthetic Surgery, Multidisciplinary Breast Clinic, Antwerp University Hospital, Antwerp, Belgium; ^10^ Department of Plastic, Reconstructive and Aesthetic Surgery, Ziekenhuis Netwerk Antwerpen, Antwerp, Belgium; ^11^ Division of Plastic and Reconstructive Surgery, Cedars Sinai Medical Center, Los Angeles, CA, United States; ^12^ Unité; Mixte de Recherche UMR 1236 Suivi Immunologique des Thérapeutiques Innovantes, INSERM and University of Rennes, Rennes, France; ^13^ Unité; Mixte de Recherche UMR-S 1140 Innovative Therapies in Haemostais, INSERM and University of Paris, Paris, France

**Keywords:** coil, COMSOL, diagnostic, iron oxide, magnetic nanoparticles, superparamagnetism

In the published article, the author Antoine A. Ruzette was erroneously excluded. The correct author list appears above.

The **Author Contributions** statement has therefore been updated as follows:

“LV: conceptualization, data curation, formal analysis, investigation, methodology, software, validation, writing–original draft, writing–review and editing, funding acquisition, project administration, resources, supervision, and visualization. AAR: formal analysis, investigation, methodology, software, writing-review and editing. VT: investigation, validation, and writing–original draft. HO: validation and writing–review and editing. YB: investigation and writing–review and editing. JC: validation and writing–review and editing. FT: investigation, supervision, validation, and writing–review and editing. CC: investigation, supervision, validation, and writing–review and editing. KU: investigation, supervision, validation, and writing–review and editing. AL: conceptualization, data curation, formal analysis, funding acquisition, investigation, methodology, project administration, resources, software, supervision, validation, visualization, and writing–review and editing.”

The authors apologize for this error and state that this does not change the scientific conclusions of the article in any way. The original article has been updated.

